# Mechanism and Improved Dissolution of Glycyrrhetinic Acid Solid Dispersion by Alkalizers

**DOI:** 10.3390/pharmaceutics12010082

**Published:** 2020-01-20

**Authors:** Luning Dong, Yaping Mai, Qiang Liu, Wannian Zhang, Jianhong Yang

**Affiliations:** 1 Department of Pharmaceutics, School of Pharmacy, Ningxia Medical University, No.1160 Shengli South Street, Yinchuan 750004, China; dln951024@163.com (L.D.); maiyp0823@163.com (Y.M.); Lqspu2011sgwt@163.com (Q.L.); 2 School of Pharmacy, Second Military Medical University Shanghai, No.800 Xiangyin Road, Shanghai 200433, China

**Keywords:** solid dispersion, alkalizers, glycyrrhetinic acid, molecular interactions, dissolution

## Abstract

The purpose of this study was to increase the dissolution of glycyrrhetinic acid (GA) by preparing ternary solid dispersion (TSD) systems containing alkalizers, and to explore the modulating mechanism of alkalizers in solid dispersion systems. GA TSDs were prepared by hot melt extrusion (HME) with Kollidon^®^ VA64 as the carrier and L-arginine/meglumine as the alkalizers. The in vitro release of the TSD was investigated with a dissolution test, and the dissociation constant (pKa) was used to describe the ionization degree of the drug in different pH buffers. Scanning electron microscopy (SEM), differential scanning calorimetry (DSC), X-ray powder diffraction (XRPD), Fourier Transform Infrared Spectroscopy (FTIR), Raman spectra, X-ray photoelectron spectroscopy (XPS), and a molecular model were used for solid-state characterizations and to study the dissolution mechanism of the TSDs. It was evident that the dissolution of GA significantly increased as a result of the TSD compared to the pure drug and binary solid dispersion. SEM, DSC, and XPRD data showed that GA transformed into an amorphous form in TSD. As illustrated by FTIR, Raman, XPS, and molecular docking, high binding energy ion-pair complexes formed between GA and the alkalizers during the process of HME. These can destroy the H-bond between GA molecules. Further, intermolecular H-bonds formed between the alkalizers and Kollidon^®^ VA64, which can increase the wettability of the drug. Our results will significantly improve the solubility and dissolution of GA. In addition, the lower pKa value of TSD indicates that higher ionization is beneficial to the dissolution of the drug. This study should facilitate further developments of TSDs containing alkalizers to improve the dissolution of weakly acidic drugs and gain a richer understanding of the mechanism of dissolution.

## 1. Introduction

At present, most widely used drugs, natural products, and drug candidates are insoluble in water, and increasing the solubility of poorly soluble drugs is an important problem for many drug molecules [1,2,3]. Several strategies have been studied to overcome this problem, such as salt formation [4,5,6], nanoparticles [7,8,9,10], inclusion complexes [11], liposomes [12,13], and amorphous solid dispersions [14,15,16,17].

Among the known technologies, amorphous solid dispersions have become one of the most effective methods of enhancing the solubility and gastrointestinal absorption of poorly soluble drugs [18,19,20]. Hot melt extrusion technology has been widely adopted because it offers continuous control and because it is solvent free and less time consuming. The drug can be dispersed in a polymer in an amorphous state, and has the advantages of higher porosity, reduced particle size, and improved wettability [21]. The polymers in solid dispersions also help to stabilize the amorphous form of the drug and prevent recrystallization [22,23]. However, one disadvantage to binary solid dispersion (BSD) is its limited solubilization capacity, especially with pH-dependent drugs. Previous studies have shown that the solubility of pH-dependent drugs can be observably improved by ternary solid dispersion (TSD) systems that incorporate pH-modifiers. McFall [24] reported that the dissolution and oral bioavailability of aripiprazole improved from the use of succinic acid as an acidifier. Tran [25] pointed out that Na_2_CO_3_, as an alkalizer to adjust the pH microenvironment, enhanced the dissolution rate of weakly acidic drugs such as aceclofenac in a solid dispersion (SD) system. Choi [26] demonstrated that by increasing solubility and improving in the vitro dissolution rate of tadalafil, tartaric acid can improve oral bioavailability and thus reduce effective drug doses. Sun [27] developed a pHM-SD system of Toltrazuril with Ca(OH)_2_ as an alkalizer, and in vitro dissolution studies indicated that the system improved the water-solubility and bioavailability compared to the pure drug. Nevertheless, most of these studies focused solely on the role of acidifiers on weakly alkaline drugs. There are few studies on using alkalizers to improve the solubility of weakly acidic drugs. In particular, there is limited research on the role of pH-modifiers as a mechanism for regulating dissolution. 

**Figure 1:**
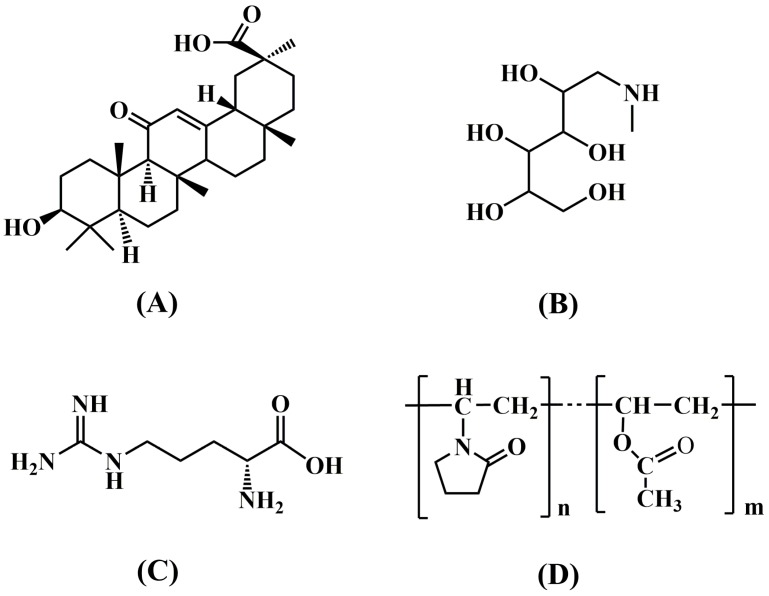
Chemical structure of glycyrrhetinic acid (**A**), meglumine (**B**), L-arginine (**C**), and Kollidon^®^ VA64 (**D**).

Glycyrrhetinic acid (GA, Figure 1A), the main bioactive component of Glycyrrhiza glabra, has been found to have pharmacological properties such as anti-inflammation, anti-virus, anti-allergy, anti-hepatotoxicity, and anti-tumor effects [28,29,30]. However, GA is insoluble in water and has pH-dependent solubility, which limits its application. Previous studies have found that BSDs make it difficult to improve the dissolution of GA effectively. The aim of this study was to prepare a SD system with the incorporation of alkalizers to improve solubility and in vitro dissolution. Further, we analyzed the modulating mechanism of alkalizers in SD systems. TSDs of GA were prepared by hot melt extrusion. The aqueous solubility and dissolution were evaluated for the pure drug and TSD, while the dissociation constant (pKa) was used to describe the ionization degree of the drug in different pH buffers. SEM, DSC, XPRD, FTIR, Raman, XPS, and a molecular model were used to perform solid-state characterizations and to study the dissolution mechanism of the TSDs. 

## 2. Materials and Methods

### 2.1. Materials

The following materials were obtained from commercial suppliers and were used as received: Na_2_CO_3_, NaOH, CaCO_3_, Mg(OH)_2_, Na_2_HPO_4_, and MgO were all obtained from Guangnuo Chemical Technology Co., Ltd. (Shanghai, China). Polyvinylpyrrolidone (Kollidon^®^ VA64, BASF, Ludwigshafen, Germany), polyethylene caprolactam-polyvinyl acetate-polyethylene glycol graft copolymer (Soluplus), poloxamer 407, and poloxamer 188 were all obtained from Fengli Jingqiu Pharmaceutical Co., Ltd. (Beijing, China). L-arginine (LA) was purchased from Zhongqin Chemical Reagent Co., Ltd. (Shanghai, China). Meglumine (MG) was purchased from Tian Run Pharmaceutical Co., Ltd. (Guangzhou, China). GA was purchased from Yuanye Bio-Technology Co., Ltd. (Shanghai, China). Affinisol was supplied by Dow Chemical (China) Co., Ltd. (Shanghai, China). Ethyl cellulose (EC) and hydroxypropyl methyl cellulose (HPMC) were purchased from Anhui Shanhe Medicinal Accessories Co., Ltd. (Huainan, China). A hydrochloric acid solution was obtained from Tianjin Damao Chemical Reagent Factory (Tianjin, China). 

### 2.2. Pre-Prescription Study

#### 2.2.1. Solubility Study of the Alkalizers and Polymers

The alkalizers and polymers listed above were screened to select components suitable for SD. Alkalizers or polymers (400 mg) were added to a centrifuge tube containing 40 mL of various media (pH 1.2, pH 4.5, pH 6.8, or pH 7.4) to prepare a 1% aqueous solution. Excess GA was added to a centrifuge tube containing each aqueous solution. The aqueous solution was vigorously vortexed and placed in a shaking water bath at 75 rpm for 72 h at 37 °C. Then, they were centrifuged at 25 °C and 8500 rpm for 10 min to separate undissolved GA. The supernatant was filtered with a 0.45 μm water filter and diluted with the corresponding pH buffers. The GA was quantified using a UV-Vis spectrophotometer (UV-1780, Shimadzu, Suzhou, China). Absorbance at 252 nm was measured with a spectrophotometer. All tests were repeated three times.

#### 2.2.2. Preparation of Solid Dispersions

According to the results of the solubility study, Mg(OH)_2_, Na_2_CO_3_, meglumine, and L-arginine were selected as alkalizers and Kollidon^®^ VA64 was selected as the hydrophilic SD carrier. All excipients were sieved through an 80-mesh standard screen and placed in a vacuum oven (DZF-6090, Qixin Scientific Instrument Co., Ltd., Shanghai, China) for 12 h at 40 °C to remove excess water. Materials were mixed evenly with a three-dimensional mixer (SYH-5, Binda Drying Granulation Equipment Co., Ltd., Hangzhou, China). Then, all samples were prepared by a 16 mm twin-screw hot melt extruder (ME-D16, Xinyite Technology Co., Ltd., Shenzhen, China) at 160 °C. Screw speed was adjusted to 80 rpm. When the extrudate was cooled to ambient temperature, it was comminuted with an experimental high-speed pulverizer until the fine powder could pass through a 200-mesh standard sieve. The sieved extrudate was stored in a brown glass bottle until further analysis. Table 1 and Table 2 show formulations for the hot melt extrusion. Finally, physical mixtures of the same ratio were prepared. All samples were stored in a desiccator before use to reduce the effects of hygroscopicity. Table 1 and Table 2 show the composition of the SD powders.

#### 2.2.3. Dissolution Study of SDs

Drug dissolution was studied with formulations at 37 ± 0.5 °C (75 rpm, paddle apparatus, AT Xtend Semi, SOTAX, Aesch, Switzerland) according to the Chinese pharmacopoeia (2015). A buffer (pH 1.2 or pH 6.8, 900 mL in each dissolution vessel) was used as the dissolution medium. Dissolution medium (10 mL) was extracted and filtered for 5, 10, 15, 20, 30, 45, 60, 90, and 120 min, and 10 mL of the corresponding fresh medium was added for compensation. Samples were determined spectrophotometrically at a wavelength of 252 nm using a Shimadzu UV-1780 dual wavelength spectrophotometer.

### 2.3. Characterization and Analysis of Optimal Formulation

According to the results of the dissolution screening test, F2, F14, and F18 were selected as the optimal formulation with a better drug release curve than the pure drug and other formulations.

#### 2.3.1. Dissociation Constant (pKa)

The measurement of pKa value of drugs by spectrophotometry is an analytical method based on the principle that the molecular state and ionic state of the substance have different absorption to a certain wavelength of light [31,32,33]. When a substance reaches the dissociation equilibrium in a solution, both the molecular and ionic states of the substance are present in the solution, and the two states have different absorptions of light of the same wavelength. Therefore, the measurement of the absorbance of the solution with a spectrophotometer is a comprehensive representation of the absorbance of molecules and ions in the solution. 

GA, BSD, meglumine ternary solid dispersion (MG-TSD), and L-arginine ternary solid dispersion (LA-TSD) samples with the same concentration were taken to prepare three solutions for testing, respectively. By adjusting the pH, they can exist in solution in different dissociation states. Then, the absorbance (*A_x_*, *A_H__M_*, and *A_M_^−^*) was measured with an ultraviolet spectrophotometer at 252 nm, and its dissociation constant (pKa) was calculated as follows:

(1) $$\mathrm{pKa}=\mathrm{pH}+\mathrm{lg}\frac{A_{x}-A_{M^{-}}}{A_{HM}-A_{x}}$$

where *A_M_^−^* is the absorbance of 100% ionic type, *A_HM_* is the absorbance of 100% molecular type, and *A_x_* is the measured absorbance of the substance solution.

#### 2.3.2. Scanning Electron Microscopy (SEM)

The surface morphology of the samples was examined with scanning electron microscopy (Carl Zeiss, Inc., Peabody, MA, USA). Each sample was sputter-coated with a gold layer beforehand to make it conductive. 

#### 2.3.3. Differential Scanning Calorimetry (DSC)

DSC (METTLER Toledo, GmbH, Zurich, Switzerland) was used to study the thermodynamic properties of the samples and their corresponding physical mixtures. Samples of about 2–4 mg were weighed and placed in a standard aluminum pan with a vent cap and sealed. The analysis was carried out at a heating rate of 10 °C/min from 25 °C to 350 °C. The DSC curve was obtained by purging at a flow rate of 50 mL/min under a dynamic nitrogen atmosphere. 

#### 2.3.4. X-ray Powder Diffraction (XRPD)

The XPRD pattern of the polymer was obtained using a Bruker D8 diffractometer (D8 Advance, Bruker AXS, Karlsruhe, Germany), and the crystal state of the GA in different samples was examined. The diffractometer was equipped with Ni-filtered Cu-Kα as an X-ray source. It was operated using a copper anode tube at a generator voltage and current of 40 kV and 40 mA, respectively. The 2-theta scan range was set from 5° to 60° at a rate of 0.02°/min. The data was further analyzed with OriginPro 8 software.

#### 2.3.5. Fourier Transform Infrared Spectroscopy (FTIR)

In order to obtain direct information about the molecular details of the drug and its interaction, FTIR spectra were obtained on a Bruker Vertex 70 spectrometer (Kennewick, WA, USA). The samples were separately dissolved in EA by KBr coating, dropped onto KBr particles, and then dried for 5 min at 50 °C to remove EA. The FTIR spectrum was obtained by scanning 128 times in the spectral range of 4000–400 cm^−1^. Deconvolution and analysis were performed using OriginPro 8 (OriginLab, Northampton, MA, USA).

#### 2.3.6. Raman Spectroscopy

Raman was used as complement to FTIR for a more direct characterization of the molecular details of the interaction between the drug and the alkalizers. Raman spectroscopy was recorded on a Renishaw (Renishaw, London, UK) inVia laser micro-Raman spectrometer using a 785 nm laser source with power of 300 mW. Samples were placed on an aluminum plate in front of a 50 × objective lens. The acquisition time for each spectrum was 10 s. A Raman spectrum was obtained in the spectral range of 3500–200 cm^−1^. The spectral data was first pre-processed using WiRE software (Wire Swiss, Zug, Switzerland) and the peak position was deconvoluted and analyzed using PeakFit 4.0 software (Systat Software, San Jose, CA, USA).

#### 2.3.7. X-ray Photoelectron Spectroscopy (XPS)

XPS (Thermo Fisher Scientific, Waltham, MA, USA) with a monochromatic aluminum Kα X-ray source was used to determine the protonation state of GA-MG and GA-LA in the SD, using MG-TSD/LA-TSD or its equimolar GA-MG/GA-LA as test samples. The data was analyzed using XPSPEAK 41 peak-fitting software.

#### 2.3.8. Molecular Modeling

Molecular modeling was used to confirm the results of FTIR, Raman, and XPS. The crystal structures of the drugs and excipients were obtained from the Cambridge Crystal Data Centre (CCDC). Deposition Number(s): glycyrrhetinic (1319339), L-arginine (1578215), and meglumine (1225097) acid Materials Studio 2017 (Accelrys, San Diego, CA, USA) was used for molecular modeling, and a COMPASS force field was used to describe intermolecular interactions.

#### 2.3.9. Molecular Docking

Geometry optimization was first performed using a Forcite module. Then, the default parameters were used for molecular docking in the Blends module. The score of the binding energy was used as the basis for selecting the best docking type.

#### 2.3.10. Molecular Dynamic Simulation

After the TSD system was constructed according to the proportion of actual formulations using the Amorphous cell module, the composite structure was optimized with the Forcite module. Then, a molecular dynamic simulation was performed on the systems in the NPT at 298 K with a time step of 1 fs to obtain the equilibrium structures. 

#### 2.3.11. Solubility and Dissolution of Optimal Formulation

For the solubility study, excess amounts of the extrudates were added to 15 mL centrifuge tubes containing different media (pH 1.2, 4.5, 6.8, and 7.4). The aqueous solution was vigorously vortexed and placed in a shaking water bath at 75 rpm for 72 h at 37 °C. Then, the samples were centrifuged at 25 °C and 8500 rpm for 10 min to obtain a supernatant. Finally, the supernatant was filtered with a 0.45 μm water filter and diluted with the corresponding pH buffer. The absorbance at 252 nm was measured by spectrophotometry. The dissolution was studied with the SOTAX dissolution tester. API, BSD, MG-TSD, and LA-TSD were placed in 900 mL of different pH buffers at 37 ± 0.5 °C to determine the drug release. We used the paddle method at 75 rpm. Media (10 mL) was extracted after 5, 10, 15, 20, 30, 45, and 60 min, and an equivalent volume of fresh media was replaced. Samples were determined spectrophotometrically at a wavelength of 252 nm using a wavelength spectrophotometer.

## 3. Results and Discussion

### 3.1. Pre-Prescription Study

#### 3.1.1. Solubility Screening 

Suitable alkalizers and polymers were screened to increase the solubility and dissolution of the drug. Specific experiments showed that all excipients, including alkalizers and polymers, did not show absorption at 252 nm in HCl (pH 1.2), pH 4.5, pH 6.8 and 7.4 buffers. 

**Figure 2:**
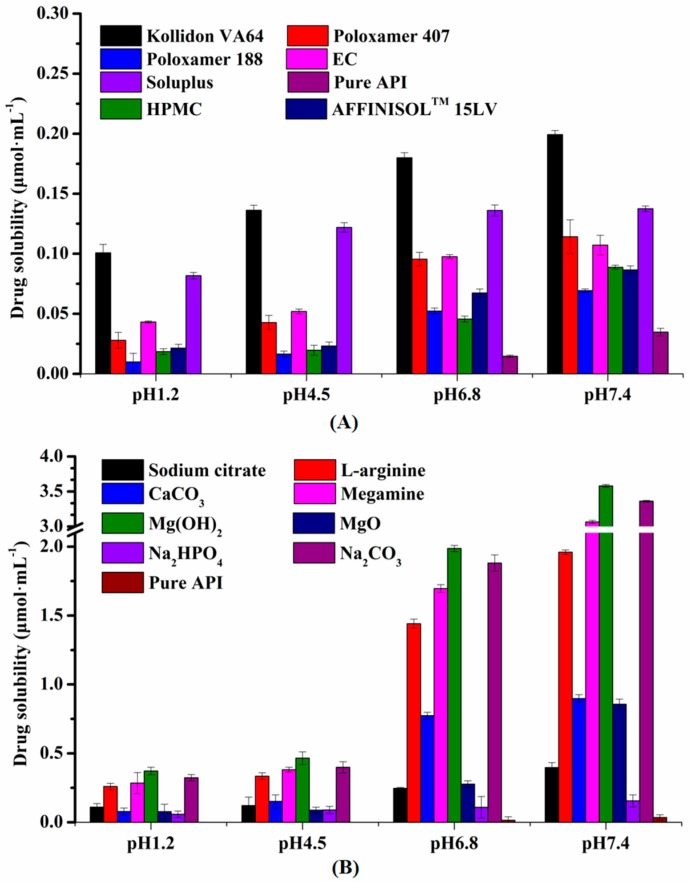
Solubility screening of polymers and alkalizers. (**A**) The solubility of glycyrrhetinic acid (GA) with various polymers in different pH buffers (*n* = 3). (**B**) The solubility of GA with various alkalizers in different pH buffers (*n* = 3).

As shown in Figure 2A, API had the highest solubility in Kollidon^®^ VA64, and it was selected as the polymer for this experiment. Moreover, the solubility of API increased significantly from the strong basicity of L-arginine, meglumine, Mg(OH)_2_, and Na_2_CO_3_ (Figure 2B). Therefore, Mg(OH)_2_, Na_2_CO_3_, meglumine, and L-arginine were selected as the alkalizers in this study. 

#### 3.1.2. Dissolution Screening of Several Formulations with Different Drug/Alkalizer/Polymer Ratios

**Figure 3:**
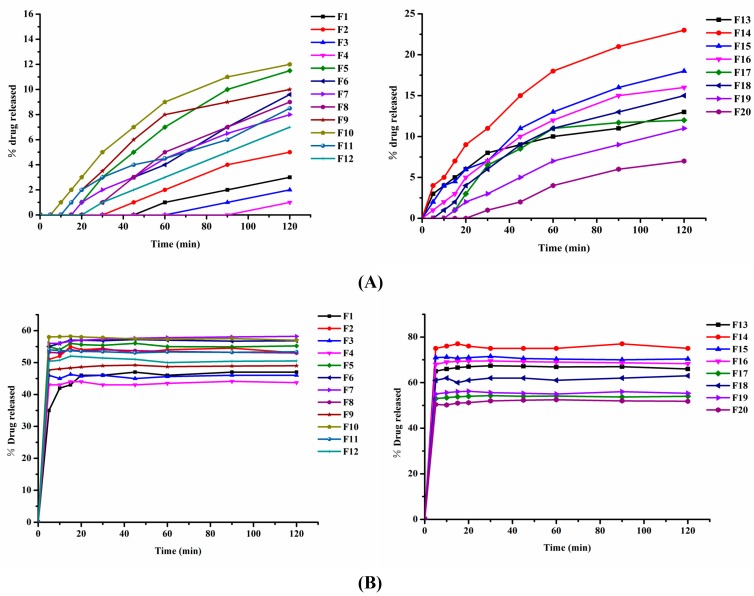
Dissolution of several formulations with different drug/alkalizer/polymer ratios. (**A**) Dissolution of F1–F20 in pH 1.2 HCl (*n* = 6). (**B**) Dissolution of F1–F20 in pH 6.8 buffer (*n* = 6).

As shown in Figure 3, F2 had a better drug release profile than the pure drug and other BSD formulations. There was no significant difference in the dissolution curve of the same proportion of formulations containing four different types of alkalizers in the pH 1.2 buffer (Figure 3A). However, the formula containing L-arginine/meglumine showed a better dissolution curve than the formula containing Mg(OH)_2_/Na_2_CO_3_ in the pH 6.8 buffer (Figure 3B). Among them, F14 and F18 had the highest level of drug dissolution among the TSDs. Therefore, F2, F14, and F18 were selected as the optimal formulations for further study. 

### 3.2. Characterization and Analysis of Optimal Formulation

#### 3.2.1. Dissociation Constant (pKa)

The Nernst–Noyes–Whitney equation [34] is often used to explain the relationship between drug dissolution and drug saturation solubility:

(2) $$\frac{dC_{b}}{\mathrm{dt}}=\frac{DS}{Vh}\left(C_{S}-C_{b}\right)$$

where *C_s_* is the saturated solubility of the drug on a solid surface, *C_b_* is the concentration of the drug in a large amount of medium, *h* is the thickness of the diffusion layer in the medium, *D* is the diffusion coefficient, *S* is the surface area of the dissolved solid, *V* is the volume of the dissolution medium, and *t* is time. It is well known that the degree of ionization of weakly acidic drugs in different pH buffers varies widely, exhibiting pH-dependent solubility. The solubility of an ionizable drug at a given pH can be expressed in terms of the Henderson–Hasselbalch equation [35]. The expression of this equation for monoacidic compounds is

(3) $$C_{S}=C_{S}{}_{0}\left[1+10^{\left(pH-pKa\right)}\right]$$

where *C_s_* is the drug solubility at a given pH, and *C_S_*_0_ is the inherent solubility of the drug.

The dissociation constant (pKa), a polar parameter of a solute with a certain degree of dissociation in aqueous solution, plays a crucial role in the solubility and dissolution of ionogenic drugs. As shown in Table 3, the pKa values of all preparations were reduced compared to the API. Among them, the pKa value of the TSD with the pH modifier decreased more significantly than the BSD and the pure drug. Further, the MG-TSD showed a lower pKa level than the LA-TSD. According to the Henderson–Hasselbalch equation, a slight shift in pKa strongly influences the aqueous solubility of drugs. A decrease in pKa can increase the *C_s_* remarkably, achieving a supersaturated state in the bulk solution and leading to increased drug dissolution [36].

#### 3.2.2. SEM

**Figure 4:**
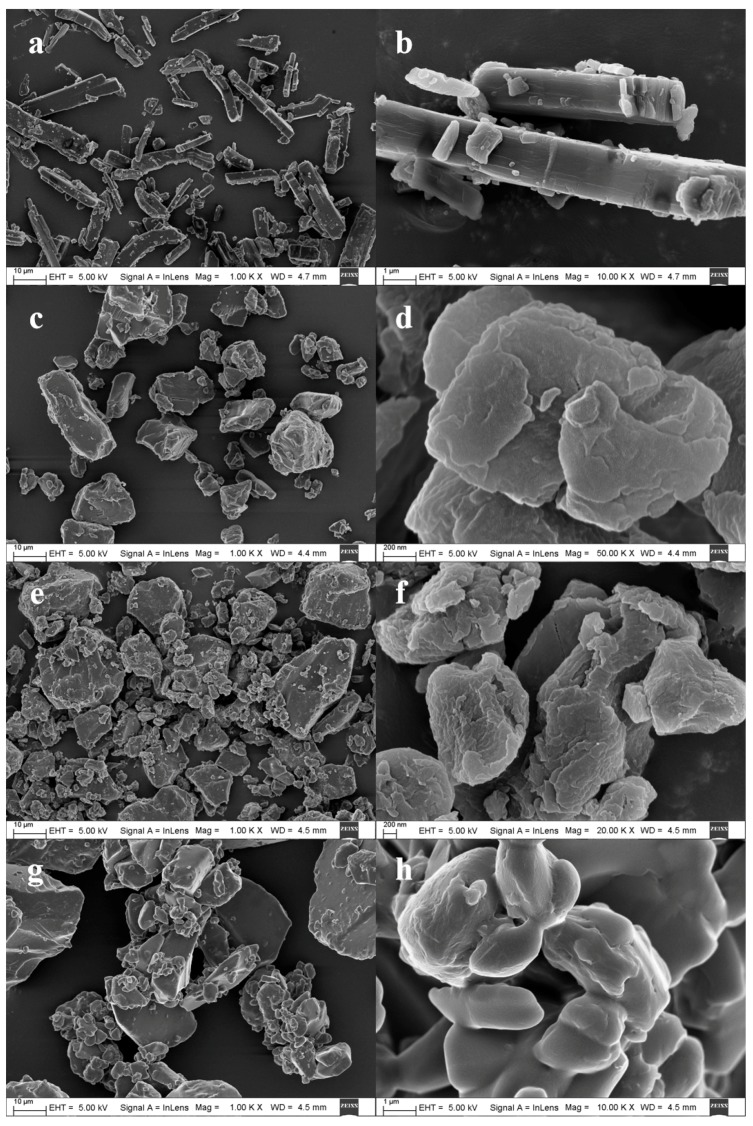
Representative SEM images of (**a**,**b**) GA, (**c**,**d**) BSD, (**e**,**f**) LA-TSD, and (**g**,**h**) MG-TSD.

SEM was used to observe the changes in the surface morphology of the materials. GA crystals with a size of 5–20 μm are clearly distributed in Figure 4a,b, in the shape of a stick cube. By contrast, we did not find any GA crystals except large particles in all solid dispersions (Figure 4c–h). These phenomena suggested that the three raw materials were intimately and homogeneously combined in the TSD particles, and that the surface of the formulation particles was covered by Kollidon^®^ VA64 macromolecules [37].

#### 3.2.3. DSC

**Figure 5:**
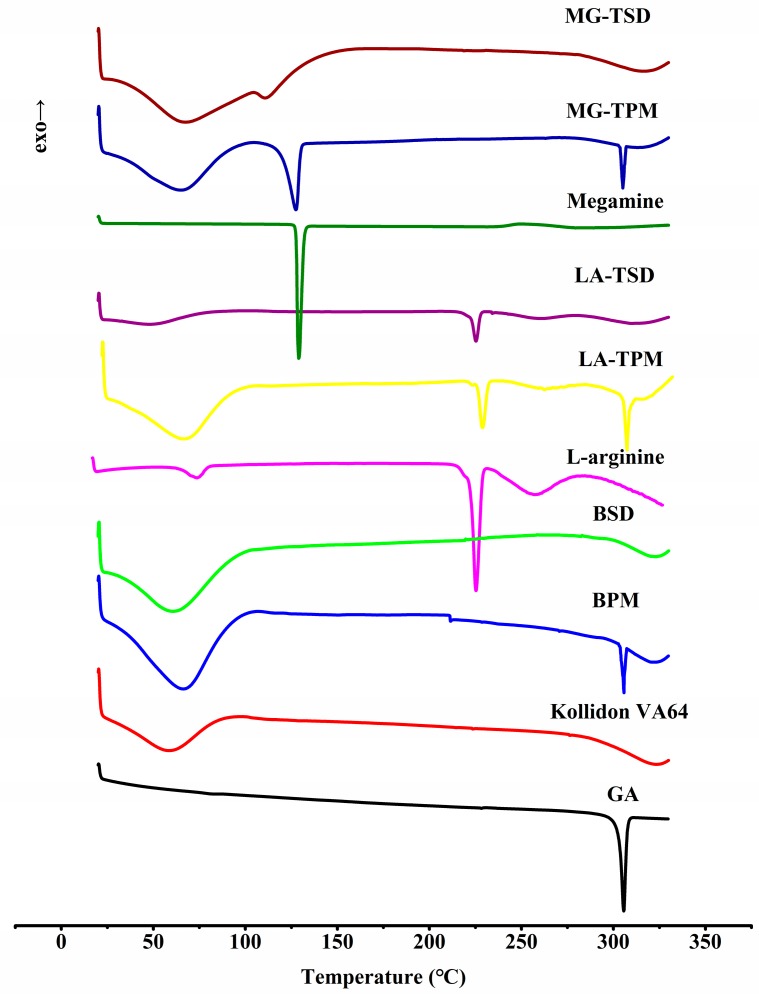
DSC thermograms of GA, Kollidon^®^ VA64, and their PMs/SDs with and without alkalizers.

In this study, DSC was used to verify the physical state of the drugs in the formulation. Figure 5 shows DSC thermograms of GA, Kollidon^®^ VA64, and their PMs/SDs with and without alkalizers. The DSC curves of pure GA, LA, and MG exhibit single endothermic peaks at 305, 228, and 129 °C, respectively, corresponding to their intrinsic melting points. By comparison, Kollidon^®^ VA64, an amorphous polymer, exhibits no melting peak in its DSC curve. The characteristic melting point peaks of GA, LA, and MG exist in all physical mixtures. As expected, no melting peak of GA was observed in any solid dispersion. The characteristic melting point peaks of LA and MG exist in their SDs, but vary via their molecular interaction with other components [25]. Taken together, these results demonstrated that the BSD and TSD systems were in amorphous forms. It should be noted that when the crystallinity of a drug is under 2%, the melting peaks of the drug cannot generally be detected with DSC [27]. For this reason, further studies using PXRD were carried out to check the crystallinity of GA in the BSD and TSD systems.

#### 3.2.4. XRPD

**Figure 6:**
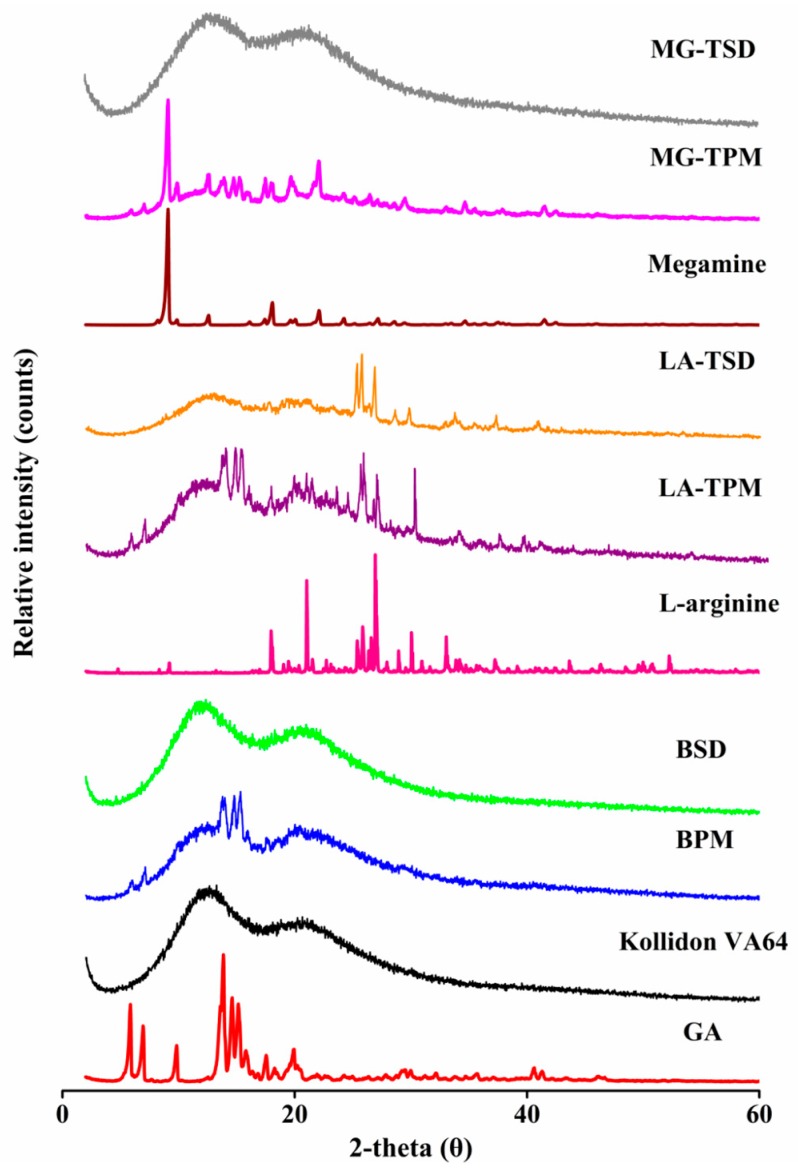
PXRD images of GA, Kollidon^®^ VA64, and their PMs/SDs with and without alkalizers.

To further confirm whether BSD and TSD systems were in amorphous forms (as suggested by the low sensitivity of DSC as mentioned above), XPRD was employed as the most convenient and direct approach [38]. XPRD images of GA, Kollidon^®^ VA64, and their PMs/SDs with and without alkalizers are given in Figure 6. The XPRD diffractogram of pure GA was highly crystalline, with many characteristic peaks. Similarly, typical high crystallinity peaks of LA and MG were observed. Kollidon^®^ VA64 has a background pattern with two very wide peaks, indicating that Kollidon^®^ VA64 exists in amorphous form. At the same time, the characteristic peaks of GA, LA, and MG were observed in their physical mixtures. The characteristic peak of GA was not observed in all SD formulations, indicating that the crystal transformation of GA occurred during hot melt extrusion. It is well known that polymers can inhibit crystallization and reduce the particle size to obtain better wettability, changing the form of the drug from crystallized to amorphous and preventing its recrystallization [22,39,40]. However, the crystal form of the drug can be further induced to change to an amorphous form with alkalizers [41]. Unexpectedly, the characteristic peak of LA was found in LA-TSD, which may be related to its stable crystallization properties (the melting temperature is 223 °C and the decomposition temperature is 244 °C). Collectively, these results demonstrate that a 100% amorphous form could be achieved through the use of amorphous Kollidon^®^ VA64 as the carrier, regardless of which alkalizers (L-arginine or meglumine) were used. Faster drug dissolution and greater solubility of the drug in an amorphous SD can be achieved by higher porosity, reduced particle size, improved wettability, and prevented recrystallization.

#### 3.2.5. FTIR

Intermolecular interactions can play an important role in the dissolution of drugs [37]. The hydrogen bond (H-bond), with moderate molecular interaction strength, is considered important for drug dissolution [42]. Existing studies have shown that the ion-pair is a relatively strong molecular force, which is aggregated by a pair of oppositely charged ions held together by coulomb attraction [43]. El Shaer utilized hydrophilic amino acids as counter ions with lipophilic indomethacin, successfully improving its solubility and dissolution profile [44]. In our study, FTIR was used to detect potential intermolecular interactions in the SD systems.

**Figure 7:**
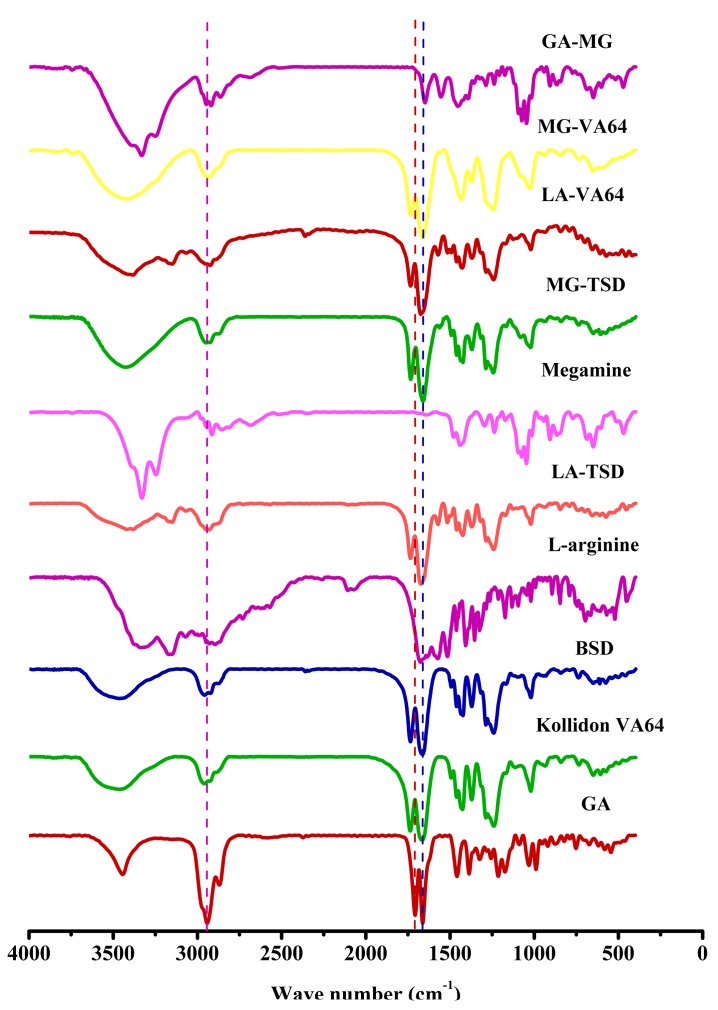
FT-IR spectra of GA, Kollidon^®^ VA64, and their PMs/SDs with and without alkalizers.

As shown in Figure 7, there are two strong peaks of GA at 1706 cm^−1^ and 1662 cm^−1^ that belong to the stretching vibration of the C=O group of the carboxylic acid and ketone segment in the GA structure, respectively. The characteristic peak of the telescopic vibration absorption of C_3_ is at 3442 cm^−1^. Kollidon^®^ VA64 contains two hydrogen-bonded receptor groups, which come from the C=O group of the pyrrolidone ring (at 1668 cm^−1^) and vinyl acetate (at 1737 cm^−1^). The spectrum of BSD is similar to that of blank Kollidon^®^ VA64, indicating that there is no significant interaction between GA and Kollidon^®^ VA64. 

**Figure 8:**
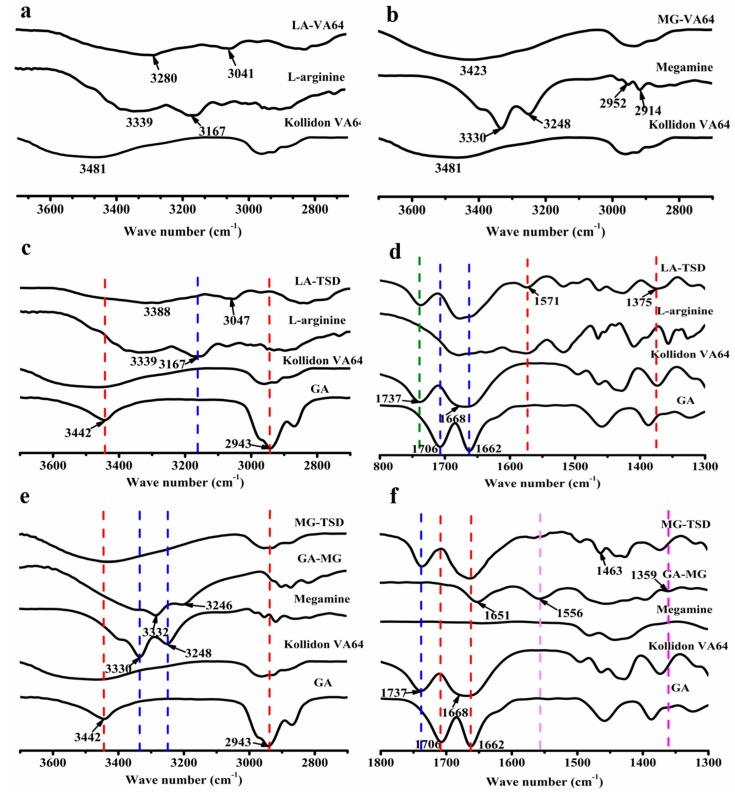
FT-IR spectra of GA, Kollidon^®^ VA64, and their SDs with and without alkalizers in a specific wavenumber range. (**a**) FT-IR spectra of Kollidon^®^ VA64, L-arginine and their extrudate in wavenumber range of 3700–2700. (**b**) FT-IR spectra of Kollidon^®^ VA64, meglumine and their extrudate in wavenumber range of 3700–2700. (**c**) FT-IR spectra of GA, Kollidon^®^ VA64, L-arginine and LA-TSD in wavenumber range of 3700–2700. (**d**) FT-IR spectra of GA, Kollidon^®^ VA64, L-arginine and LA-TSD in wavenumber range of 1800–1300. (**e**) FT-IR spectra of GA, Kollidon^®^ VA64, meglumine, GA-MG extrudate and MG-TSD in wavenumber range of 3700–2700. (**f**) FT-IR spectra of GA, Kollidon^®^ VA64, meglumine, GA-MG extrudate and MG-TSD in wavenumber range of 1800–1300.

As shown in Figure 8a,b, the peak near 3339 cm^−1^ is due to the N-H tensile vibration of the primary amine group in LA [45], which is located at 3280 cm^−1^ in the LA-VA extrudate. The band at 3330 cm^−1^ corresponds to the secondary amine group in MG [46], which vanished from the curve of the MG-VA extrudate. In addition, the absorption peak of Kollidon^®^ VA64 at 3481 cm^−1^ was shifted to 3423 cm^−1^. A wide range of wavenumber shifts indicate that an ionic hydrogen bond formed between the alkalizers and Kollidon^®^ VA64, with the (-NH_2_/-NH-) group protonated to (-NH_3_^+^/-NH_2_^+^-) [47,48,49]. The protonated amino group in alkalizers is the hydrogen donor [25,50], while the C=O group of pyrrolidone in Kollidon^®^ VA64 is a stronger hydrogen bond acceptor than the acetic acid group [51,52].

Two significant changes were found in the FTIR spectra of LA-TSD (Figure 8c,d): The C_3_ tensile vibration band of GA at 3442 cm^−1^ disappeared and there were two new peaks at 1571 cm^−1^ and 1375 cm^−1^, attributed to the asymmetric (V^as^_COO_^−^) and symmetric stretching vibration (V^s^_COO_^−^) of the -COO^−^ group [53]. Similarly, according to Figure 8e,f, two new peaks—the asymmetric (V^as^_COO_^−^) and symmetric stretching vibration (V^s^_COO_^−^) of the -COO^−^ group—appeared at 1566 cm^−1^ and 1359 cm^−1^, separately [53]. Additionally, the C_3_ tensile vibration band of GA vanished from the curve of MG-TSD. These results indicated that ion pair complexes formed from the strong ionic interaction between the GA and the alkalizers, with the -COOH group of GA deionized to -COO^−^ [45,54,55,56,57,58,59]. 

These results suggest that Kollidon^®^ VA64 relies on hydrogen bonds to interact with alkalizers, and GA interacts with alkalizers to form ion-pair complexes through strong electrostatic attraction. This may be an important reason for the increase in the dissolution of GA.

#### 3.2.6. Raman Spectroscopy

**Figure 9:**
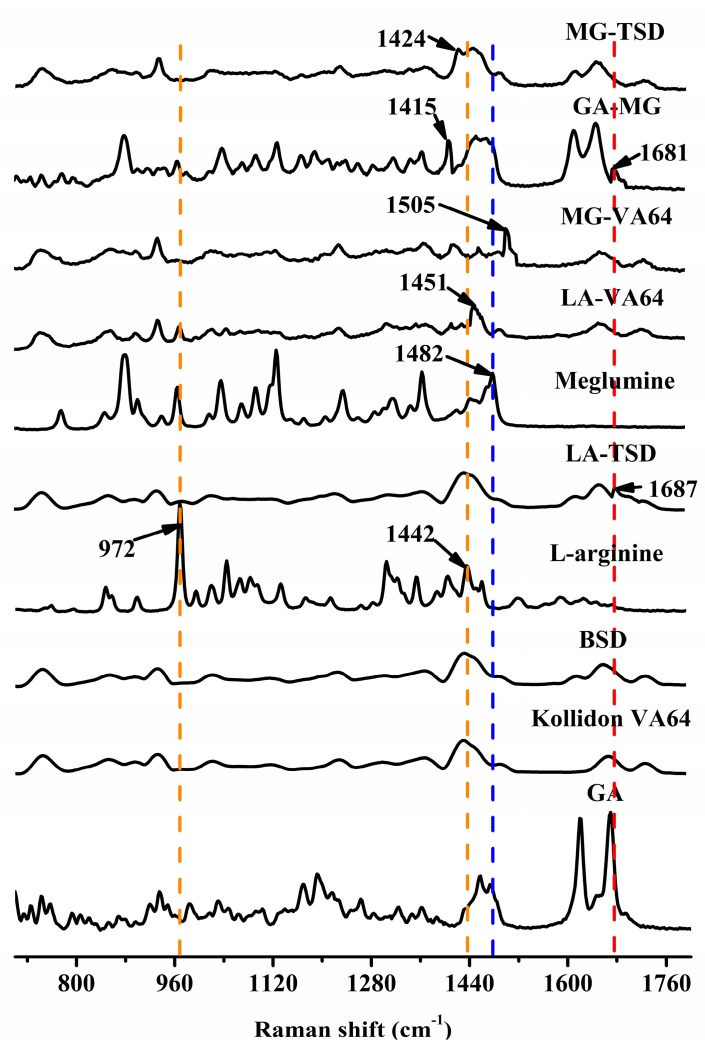
Raman spectra of GA, Kollidon^®^ VA64, and their PMs/SDs with and without alkalizers in the wavenumber range of 700 to 1800 cm^−1^.

As a supplement to FTIR, Raman spectroscopy further confirmed the ion pair effect between the drugs and alkalizers. The Raman infrared spectra are shown in Figure 9. The stronger band at 972 cm^−1^ and the weaker bands at 846 and 898 cm^−1^ are attributed to the deformation of the guanidine group in GA. Thus, the bands at 1653, 1569, 1444, and 1163 cm^−1^ belong to different deformation modes involving the N–H bond. Aliaga reached a similar conclusion by studying the surface-enhanced Raman scattering of arginine [60]. Furthermore, the peak of the primary amine group of LA (1442 cm^−1^) in the LA-VA64 extrudate appears at 1451 cm^−1^, and the peak of the secondary amine group of MG (1482 cm^−1^) in MG-VA64 extrudate is located at 1505 cm^−1^. The displacement of a larger wavenumber indicates that there is a hydrogen bond force between Kollidon^®^ VA64 and the alkalizers. The peaks at 1415 cm^−1^, 1424 cm^−1^, 1681 cm^−1^, and 1687 cm^−1^ correspond to the asymmetric and symmetric stretching vibrations of the -COO^−^ group. This is consistent with previous reports [60,61]. These results indicated that ion pair complexes formed between alkalizers and the deprotonated carboxyl group in GA. 

#### 3.2.7. XPS

**Figure 10:**
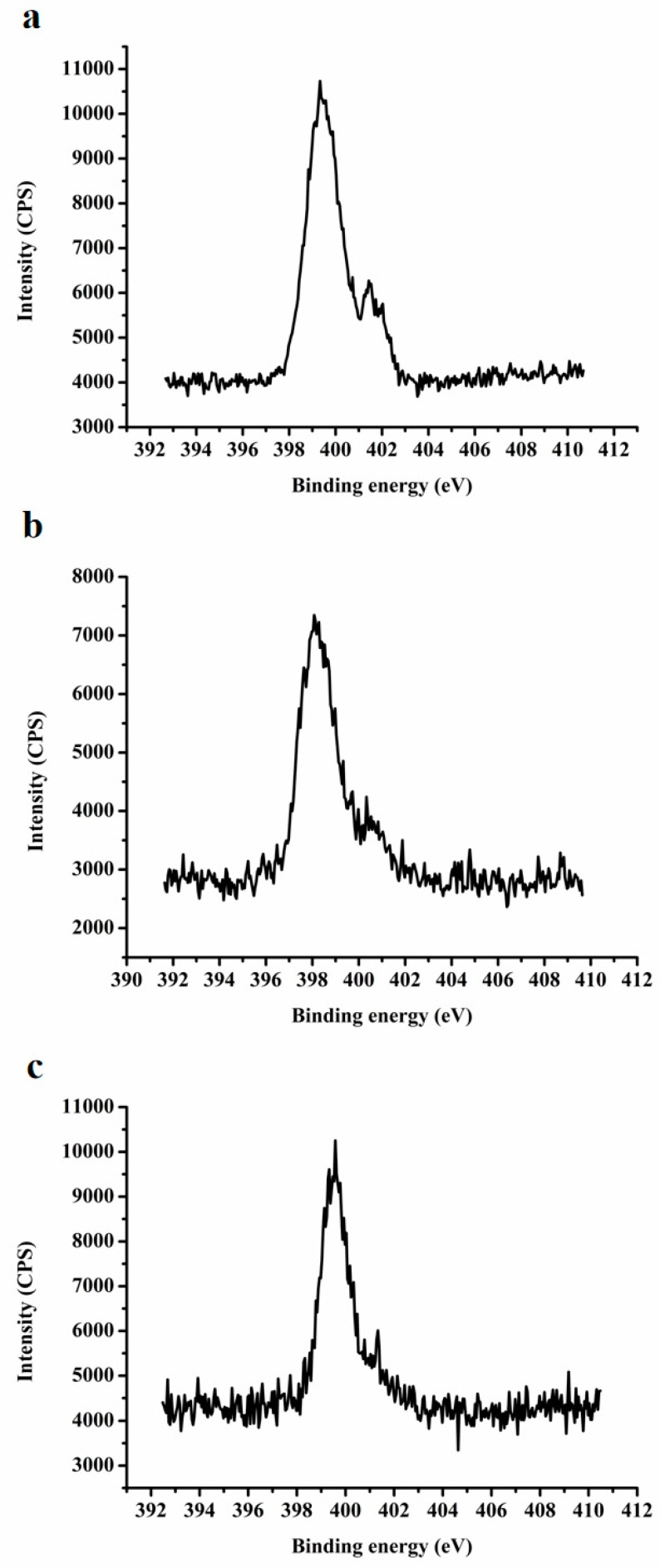
Spectrum of N1s in XPS of (**a**) LA-TSD, (**b**) GA-MG, and (**c**) MG-TSD.

XPS analysis is a highly sensitive tool. It is usually used to study the valence state and surface composition of materials [62]. In this study, XPS, as a supplement to the FTIR and Raman spectra, provides more direct evidence for the existence of ion pair complexes formed between alkalizers and the deprotonated carboxyl group in GA. Figure 10 shows the spectra of N1s in the XPS of LA-TSD, GA-MG, and MG-TSD. First, two X-ray photoelectron emission peaks were found at 401.43 and 399.33 eV of the LA-TSD spectrum. They are attributed to protonated (NH_3_^+^) and free -NH_2_ groups, respectively [63,64]. As suggested by Huang, the protonated (NH_3_^+^) has higher electron binding energy than before [65]. The peak of the N-H on the guanidine group in LA is difficult to observe, and it may be masked by the peak of -NH_2_, insofar as the two peaks appear in almost the same position. The N1s peaks of the secondary amine groups and protonation (-NH_2_^+^) have been reported in previous studies, and these are consistent with the peaks at 398.08 eV and 400.33 eV observed in the GA-MG extrudate [66]. Finally, the N1s peak of 399.58 eV and the weak peak of 401.28 eV in the XPS spectrum of MG-TSD correspond to the -NH- group and the protonated -NH_2_^+^ group, respectively. The results of XPS thus further confirmed the existence of ion-pair complexes with GA-alkalizers. Further studies using a molecular model were carried out in order to explain the possible molecular mechanism by which the ion pair effect in SDs enhances drug dissolution more clearly.

#### 3.2.8. Molecular Docking

**Figure 11:**
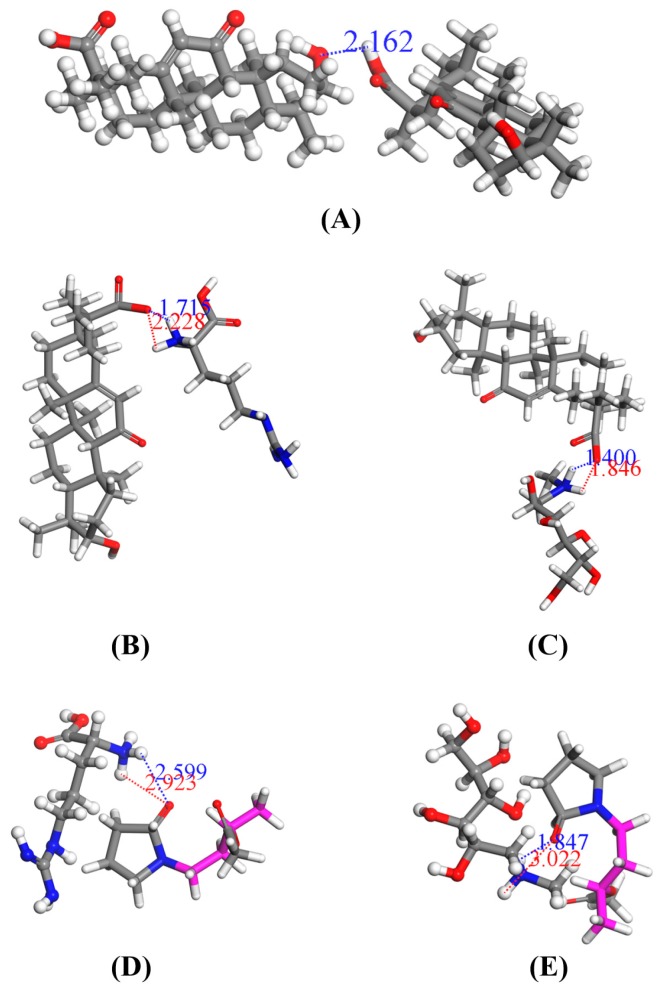
Optimal molecular docking conformation. (**A**) GA with GA, (**B**) GAH¯ with MGH^+^, (**C**) GAH¯ with LAH^+^, (**D**) Kollidon^®^ VA64 with MGH^+^, and (**E**) Kollidon^®^ VA64 with LAH^+^.

Molecular docking is often used to explain potential intermolecular interactions [54]. The use of molecular docking in this article explains how the ion pair complexes improve the dissolution rate and degree of GA in solid dispersion systems. Figure 11 shows the optimal molecular docking conformation between GA and GA, GA and alkalizers, and alkalizers and Kollidon^®^ VA64. The H-bond distance between the two GA molecules is 2.162 Å and the binding energy is 10.612 kcal/mol. The distances between -COOH^−^ of GAH^+^ and -NH_3_^+^- of MGH^+^ were 1.400 and 1.846 Å, respectively. Similarly, the distances between -COOH^−^ of GAH^−^ and (-NH_2_^+^-) of LAH^+^ were 1.715 and 2.228 Å, respectively. In addition, the binding energies of GA with MG/LA are −24.956 and −15.267 kcal/mol, respectively. In the same way, the distances between (-NH_3_^+^^−^) of MGH^+^ and Kollidon^®^ VA64 were 1.847 and 3.022 Å, respectively, while the distances between (-NH_2_^+^-) of LAH^+^ and Kollidon^®^ VA64 were 2.599 and 2.923 Å, respectively. In addition, the binding energies of Kollidon^®^ VA64 with MGH^+^ and Kollidon^®^ VA64 with LAH^+^ were −3.358 and 26.894 kcal/mol, respectively.

Intermolecular hydrogen bonds often form between glycyrrhetinic acid molecules through their carboxyl groups and hydroxyl groups, due to the strong electronegativity of oxygen atoms. In most cases, shortening the bond length can strengthen the bond [67]. The intermolecular H-bond is thought to be responsible for the poor solubility of drugs [68]. According to the results of molecular docking, shorter bond distance and smaller binding energy mean that there is a strong hydrogen bond between GA molecules, which may be a potential factor to limit the dissolution of GA. From an energy point of view, the binding energy of GA-alkalizer ion complexes is much less than that of GA molecules, which indicates the interaction between alkalizers and GA is stronger than that between GA molecules. This stronger intermolecular interaction destroys the stable crystal structure of GA, which is the inherent reason for the increased solubility of GA. Similarly, the binding energy of the GA-MG ion pair is lower than that of the GA-LA ion pair, and the hydrogen bond of the MG-VA extrudate is stronger than that of LA extrudate [68]. This explains why the dissolution of MG-TSD is higher than that of LA-TSD. Overall, the combined observations of molecular docking indicate that the ion-pair complexes improve the solubility and dissolution of GA by destroying the stable H-bond between GA molecules and strengthening the H-bond interaction with Kollidon^®^ VA64. 

#### 3.2.9. Molecular Dynamic Simulation

**Figure 12:**
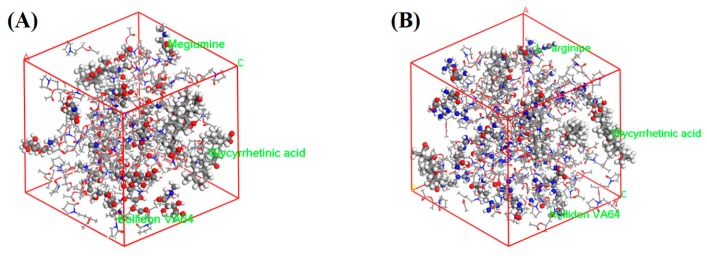
Snapshots of (**A**) MG-TSD and (**B**) LA-TSD systems at the end of the molecular dynamics simulation.

A molecular dynamics simulation was used to monitor and evaluate the conformational behavior of the TSD systems, and to validate the outcomes of the molecular docking. Snapshots of SD systems loading MG and LA are shown in Figure 12. The diffusion coefficient was used to represent the lateral movement of the drug molecules, and the cohesive energy density was used to measure the intensity of the interaction of the ion pair complexes [69]. The diffusion coefficients of MG-TSD and LA-TSD were 4.52 × 10^−11^ and 3.68 × 10^−^^11^ m^2^/s, respectively. This is consistent with the phenomena observed in the dissolution process in vitro, indicating that the diffusion of GA molecules in MG-TSD was faster. The cohesive energy density of MG-TSD and LA-TSD was 4.63 × 10^−8^ and 3.31 × 10^−8^ J/m^3^, respectively, indicating that there is a stronger intermolecular interaction in MG-TSD. Additionally, this intermolecular force could prevent the drug from spontaneously aggregating for a long time.

#### 3.2.10. Solubility and Dissolution of Optimal Formulation

**Figure 13:**
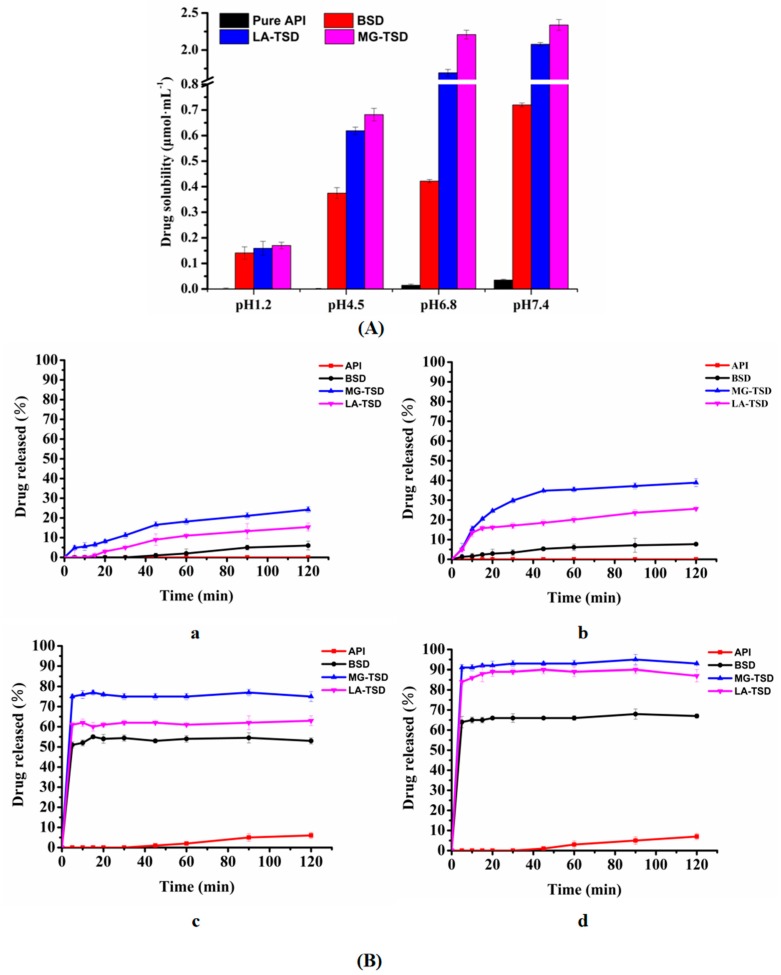
Solubility and dissolution of optimal formulations. (**A**) Solubility of GA, BSD, LA-TSD, and MG-TSD in HCl and different pH buffers (*n* = 3). (**B**) Dissolution of GA, BSD, LA-TSD, and MG-TSD in (**a**) HCl (pH 1.2), (**b**) pH 4.5, (**c**) pH 6.8, and (**d**) pH 7.4 buffers (*n* = 6).

The aqueous solubility and dissolution were evaluated for the pure drug and SDs. The solubility and dissolution of the optimal formulations in different pH buffers are shown in Figure 13. As expected, all formulations showed higher solubility than the API. There was little difference between BSD and TSD in HCl (pH 1.2), but the solubility of TSD was higher than that of BSD in the pH 4.5 buffer, in which MG-TSD was better than LA-TSD. In addition, the same trend was observed in the pH 6.8 and pH 7.4 buffers.

The in vitro dissolution test was performed for API (pure), BSD, LA-TSD, and MG-TSD in HCl (pH 1.2), pH 4.5, pH 6.8, and pH 7.4 buffers. All formulations showed higher dissolution rates and better dissolution levels than those of GA (Figure 13B). The TSD had a faster dissolution rate than the pure drug and BSD. The extent of this enhanced dissolution depended on the type of alkalizer. A moderate increase in the dissolution rate was attributed to LA, while the maximum increase in the drug release rate was attributed to MG in the HCl (pH 1.2), pH 4.5, pH 6.8, and pH 7.4 buffers. The dissolution from MG-TSD was 24.2, 38.9, 12.5, and 13.3-fold greater than that of GA in the pH 1.2, pH 4.5, pH 6.8, and pH 7.4 buffers, respectively.

In general, TSD systems containing alkalizers could enhance the dissolution of GA with the following mechanisms. First, GA was dispersed in TSD in an amorphous state, with the advantages of reducing the particle size and improving wettability, facilitating the dissolution of the drug [21]. In addition, the lower pKa value indicates that the drug in the TSD has a higher degree of ionization, and that it dissolved by achieving supersaturation in the bulk solution [36]. Most importantly, the stable H-bond between the GA molecules was destroyed by the GA-alkalizer ion pair complexes with lower binding energy. Moreover, the wettability of the drug increased from the H-bond between the alkalizers and Kollidon^®^ VA64. These results show that the intermolecular interaction in SD systems is important for improving the solubility and dissolution of GA [68].

## 4. Conclusions

The preparation of TSDs containing alkalizers is a useful method for increasing the dissolution of an ionizable drug like GA. LA and MG in SD systems significantly increased the dissolution of GA in different pH media. DSC and XPRD showed that the crystallinity of GA became amorphous in the TSD. The existence of GA-alkalizer ion pair complexes in the TSD was shown with FTIR, Raman, and XPS. The action of the ion pairs and the changes to the drug crystallinity from the alkalizers were the major contributing factors to the enhanced dissolution of GA in SDs containing alkalizers. In addition, the lower pKa value of TSDs helped the drug to achieve a supersaturated state in the bulk solution—another important factor for the increased dissolution of GA. However, GA use as a drug is also limited by its powerful mineralocorticoid activity (by blocking 11 type 2 steroid dehydrogenases and binding to mineralocorticoid receptors). Next, the study of GA’s pharmacological properties and in vivo bioavailability will be our focus.

## Figures and Tables

**Table 1:** Formulation compositions of solid dispersion (SD) powders (F1–F12).

Formulation	GA (g)	pH Modifier		Kollidon^®^ VA64 (g)	Ratio	Total (g)
		Mg(OH)_2_ (g)	Na_2_CO_3_ (g)			
F1	5	-	-	75	1:0:15	80
F2	8	-	-	72	1:0:9	80
F3	16	-	-	64	1:0:4	80
F4	32	-	-	48	2:0:3	80
F5	6	6	-	48	1:1:8	60
F6	6	12	-	42	1:2:7	60
F7	6	18	-	36	1:3:6	60
F8	12	18	-	30	2:3:5	60
F9	6	-	6	48	1:1:8	60
F10	6	-	12	42	1:2:7	60
F11	6	-	18	36	1:3:6	60
F12	12	-	18	30	2:3:5	60

**Table 2:** Formulation compositions of SD powders (F13–F20).

Formulation	GA (g)	pH Modifier		Kollidon^®^ VA64 (g)	Ratio	Total (g)
		Meglumine (g)	L-arginine (g)			
F13	6	6	-	48	1:1:8	60
F14	6	12	-	42	1:2:7	60
F15	6	18	-	36	1:3:6	60
F16	12	18	-	30	2:3:5	60
F17	6	-	6	48	1:1:8	60
F18	6	-	12	42	1:2:7	60
F19	6	-	18	36	1:3:6	60
F20	12	-	18	30	2:3:5	60

**Table 3:** pKa values of GA, binary solid dispersion (BSD), meglumine ternary solid dispersion (MG-TSD), and L-arginine ternary solid dispersion (LA-TSD) obtained from Equation (1).

NO.	pH	GA	BSD	MG-TSD	LA-TSD
1	6.5	7.17	7.02	6.42	6.72
2	7.0	7.18	7.01	6.44	6.73
3	7.5	7.17	7.07	6.44	6.71
4	8.0	7.16	7.09	6.43	6.73
Average value of pKa		7.17	7.05	6.43	6.72

## Abbreviations

SEM	Scanning electron microscopy
DSC	Differential scanning calorimetry
XRPD	X-ray powder diffraction
FTIR	Fourier transform infrared spectroscopy
XPS	X-ray photoelectron spectroscopy
EC	Ethyl cellulose
HPMC	Hydroxypropyl methyl cellulose
HME	Hot melt extrusion
LA	L-arginine
MG	Meglumine
GA	Glycyrrhetinic
SD	Solid dispersion
BSD	Binary solid dispersion
TSD	Ternary solid dispersion
LA-TSD	L-arginine ternary solid dispersion
MG-TSD	Meglumine ternary solid dispersion
BPM	Binary physical mixture
LA-TPM	L-arginine physical mixture
MG-TPM	Meglumine physical mixture
LA-VA	L-arginine-Kollidon^®^ VA64 extrudate
MG-VA	Meglumine-Kollidon^®^ VA64 extrudate
GA-MG	Glycyrrhetinic-Meglumine extrudate
